# Uncovering psychological mechanisms mediating the effects of drugs: some issues and comments using the example of psychedelic drugs

**DOI:** 10.1007/s00213-020-05703-9

**Published:** 2020-11-05

**Authors:** Samuli Kangaslampi

**Affiliations:** Faculty of Social Sciences/Psychology, Tampere University, FI-33014 Tampere, Finland

**Keywords:** Psychopharmacology, Mediation analysis, Mechanism of action, Mechanism of change, Psychedelics

## Abstract

Researchers have begun efforts to uncover the psychological mechanisms by which psychedelic drugs may have beneficial effects on long-term outcomes in some circumstances. The approaches several recent publications on the topic have taken to analyze such mechanisms have some pitfalls and limitations. Based on the rich literature on mechanisms and mediation analysis in psychological science, I comment on five particular issues: (1) Separating mediating and moderating factors, (2) problems inherent in using cross-sectional data, (3) statistical methods in mediation analysis, (4) assumptions and limitations inherent in traditional mediation analysis, and (5) criteria beyond mediation to establish a mechanism. Suggested practices for future research on the psychological mechanisms through which drugs have their effects are presented.

A number of recent publications have explored the possible psychological mechanisms through which classic psychedelics might have effects on final outcomes such as depression and anxiety (Davis et al. 2020; Roseman et al. 2018; Zeifman et al. 2020), well-being (Roseman et al. 2019), positive mood (Forstmann et al. 2020), narcissism (van Mulukom et al. 2020), or partner violence (Thiessen et al. 2018). Such work may be informative and important for understanding and maximizing the therapeutic potential of some of these substances. However, the approaches several of these publications have taken to analyzing mechanisms and paths of effects have some pitfalls and limitations that should be borne in mind when considering their findings and planning future studies on the topic. Statistical mediation and mechanisms through which effects travel have been studied extensively in psychological science. Based on lessons learned and criticisms presented in especially social psychological and psychotherapy research, I comment on five issues related to the way psychological mechanisms of psychedelics are beginning to be studied, together with some recommendations for future research. I focus on studies on classic psychedelics as a recent, relevant example, but these observations extend to all studies of the psychological mechanisms through which drugs have effects.

First, it would be helpful for authors to distinguish unambiguously whether they are attempting to elucidate mechanisms or pathways that transmit effects on final outcomes, typically through mediation analysis, the boundary conditions and contingencies of effects on final outcomes, typically through moderation analysis, or something else entirely (Hayes and Rockwood 2017; Kraemer et al. 2002). Moderation and mediation analyses do not always differ much in practical statistical implementation. But the arguments they set forth, the hypotheses they test, and the practical implications their results may have are very different. Some recent publications on psychedelics use the term *mediation* in an atypical way (Netzband et al. 2020; Roseman et al. 2018, 2019). A mediation model is at heart “a set of two or more causal events chained together in sequence of the form X ➔ M ➔ Y. So, by definition, mediator variable M must be causally located between X and Y. It must be affected by X, and it in turn must affect Y.” (Hayes and Rockwood 2017, p. 40). Mediation is about effects being transmitted through a suggested mechanism. Factors such as “therapeutic alliance, a therapeutic intention and setting and a willingness to surrender” (Roseman et al. 2019, p. 1084), or “‘context’ and particularly ‘environment’” (Roseman et al. 2018, p. 7) are generally not affected by *X* (here, ingesting a psychedelic) and are typically pre-determined, so they cannot be mediators of the drug’s effects on some final outcome *Y*. Such contextual factors may well, however, *moderate* the effects of a drug on some final outcomes (Kazdin 2007). Notably, some psychological factors could do both. For instance, it is plausible that *baseline levels* of openness could moderate the effects of psychedelics, but some research has also suggested that psychedelic use could lead to increases in openness. Such *changes* in openness may then mediate effects on other outcomes such as mental health or well-being.

Especially in the context of psychedelics, it would probably also be helpful to distinguish between acute and post-acute mediators of effects on final outcomes. An example of an acute mediator could be whether (or the extent to which) a person experiences an emotional breakthrough under the influence of a psychedelic drug. An example of a post-acute mediator could be increased social connectedness one week later as a result of taking a psychedelic drug. We might study the mediating effects of either or both of these factors on improved well-being one month after taking the drug, or indeed the putative causal chain unfolding over time from cause (taking the drug) to acute mediator (emotional breakthrough some hours later) to post-acute mediator (increased social connectedness one week later) to final outcome (well-being one month later).

Second, mediation analyses are problematic and provide limited evidence if they are based on fully or partially cross-sectional data (Maxwell et al. 2011). With one point of measurement, it is very difficult to make credible claims about directions or paths of effects, or indeed about whether changes occurred at all. Having two points of measurement, as in pre-post designs, or asking participants retrospectively to provide two assessments of their state before and after taking a drug (as in, e.g., Davis et al. 2020), though better for demonstrating changes, still provides no evidence about whether changes in a (post-acute) mechanism preceded or lead to a change in an outcome or vice versa. As a result, in several of the published analyses, the possible direction of influence, if any, between outcome and proposed mechanism could just as well be the opposite, e.g., positive mood to social connectedness (Forstmann et al. 2020), reduced depression/anxiety to increased psychological flexibility (Davis et al. 2020), or narcissism to reduced connectedness and affective empathetic drive (van Mulukom et al. 2020). Further, fully cross-sectional mediation analyses may result in highly biased estimates of effects, with the direction of the bias impossible to determine (Maxwell et al. 2011). If researchers wish to study mechanisms with cross-sectional data, they should demonstrate great restraint in how they frame their questions and interpret their findings. Mediation analyses are inherently causal in their claims (Nguyen et al. 2020), with typical graphs of mediation models explicitly claiming directions for effects. If only fully or partially cross-sectional data is available, it may be prudent to refrain from formal mediation analyses and instead simply study and speak of associations. When researchers intend to make a solid claim of mediation of effects over time, so that *X* first caused (changes in) *M*, which then caused changes in *Y*, they will need to include at least three points of measurement and conduct their analyses accordingly.

Third, some issues related to the statistical implementation of mediation analyses should be pointed out. Researchers should refrain from claiming mediation and especially “full” mediation (as in Davis et al. 2020) based on the fact that, with the addition of an indirect mediated path, the statistical significance of a remaining direct path disappears (typically, changing from *p* < .05 to *p* > .05). Such a change in significance can result from a minuscule change in the estimate for the effect (as in Thiessen et al. 2018), a rather spurious criterion. If researchers are specifically interested in whether a direct effect exists in addition to an indirect one, a different decomposition of the total effect provides a more straightforward answer (Nguyen et al. 2020).

Typically, mediation analyses focus on the strength and statistical significance of the indirect path from the proposed causal variable *X* (e.g., ingesting or not ingesting a drug) on the outcome *Y*, such as well-being, via the proposed mediating variable *M*. Many statistical procedures for carrying out this examination have been presented over the years that perform somewhat differently when the available data is not perfect (see, e.g., Hayes and Scharkow 2013; MacKinnon et al. 2002). The Sobel (1982) test used by Thiessen et al. (2018) is low-powered and best avoided. Generally, researchers should account for the inherently non-normal distribution of a product of coefficients by using bootstrap methods for significance testing or constructing confidence intervals for the indirect effect (Hayes and Scharkow 2013).

Using simultaneously evaluated structural equation modeling (SEM) for mediation analysis, as some of the publications do, rather than multiple linear regressions, may encourage process and model-oriented thinking (Pek and Hoyle 2016). All analyses in these publications used observed variables, however. In SEM, the use of latent variables to account for measurement error would reduce (typically downward) bias in estimates of the indirect effect and risk of type I errors, at the expense of reduced statistical power and precision (Ledgerwood and Shrout 2011), and should be encouraged when sample sizes permit. The popular *lavaan* package in the R programming language (Rosseel 2012) implements modern approaches to SEM with latent variables, with maximum likelihood estimation as the standard option. Notably, maximum likelihood estimation, as used by Forstmann et al. (2020), supposes multivariate normality in the included variables, but non-normality in variables can be addressed by using weighted least squares estimators, as Davis et al. (2020) did, or robust methods (Rosseel 2012). In any case, researchers should consider credible alternative models and present model comparisons, especially when studying more complex models such as those proposed by van Mulukom et al. (2020) and Forstmann et al. (2020).

Fourth, regardless of exact statistical methods used, researchers should keep in mind that the so-called traditional approach to mediation analysis makes important assumptions about the nature of the variables studied and their relations (Kraemer et al. 2002; Nguyen et al. 2020; Preacher 2015). Besides linear relations throughout, the main assumptions of concern are, first, no moderating effect of *X* on the *M* ➔ *Y* relation, and second, no unaccounted for confounding in the *M* ➔ *Y* relation. Considering the first in relation to psychedelics, it is not impossible, for example, that the effect of a mediator such as change in openness on a long-term outcome could depend on the treatment and could be different for those who received a psychedelic and those who received a placebo. This would be an example of an interaction that the standard approach does not consider, resulting in interaction effects being subsumed into the direct effect (Muthén and Asparouhov 2015). As for the second condition, in controlled research, levels of the treatment variable are randomly assigned, which removes most concerns about confounding in *X* ➔ *M* and *X* ➔ *Y*. However, the mediating variable typically cannot be manipulated. Thus, potential confounding in the *M* ➔ *Y* relation remains, due to omitted variables that might affect both *M* and *Y* or correlate with *M* (such as other omitted mediators) (Bullock et al. 2010; Pek and Hoyle 2016). This results in bias, typically overstating the degree of mediation (Bullock et al. 2010). The problem of confounding factors is naturally even more pronounced in non-randomized designs and models that incorporate several mediators in sequence, such as the analyses by Forstmann et al. (2020) and van Mulukom et al. (2020).

Researchers should explicitly acknowledge these assumptions and consider whether they are justified. The effects of deviations from these assumptions may be assessed by sensitivity analyses and mitigated by some statistical tools (MacKinnon and Pirlott 2015; Preacher 2015). Overall, researchers might find it worthwhile to move on from the traditional approach to frame and carry out their analyses using the *causal mediation analysis* framework, which also encourages explicit causal thinking and specification of which effects exactly we are pursuing in a model-free manner (Imai et al. 2010; Nguyen et al. 2020).

Finally, “Mediation is to mechanism what correlation is to cause.” (Tryon 2018; p. 626). Demonstrating that a variable mediates the effects of a drug on some outcome is not all it takes to prove that the process this variable is taken to represent is a mechanism that truly accounts for and transmits (some of) the drug’s causal effects. Other important criteria to consider have also been presented (e.g., Kazdin 2007). They include providing (preferably experimental) evidence of causality in the mechanism to outcome relation and a detailed explanation of *how*, through which steps, this mechanism could transmit effects on the outcome in question, as well as demonstrating a specific and consistent role for the mechanism over several studies. More widely, the search for psychological mechanisms should not be ad hoc in nature but driven by psychological theories or previous empirical findings. This also means preregistered, hypothesis-testing, and exploratory, hypothesis-generating, analyses should be clearly and explicitly separated. Forstmann et al. (2020) and van Mulukom et al. (2020) should be commended for committing to preregistration, even though both studies deviated from the preregistration to some extent in their analyses.

It is early days in determining the psychological mechanisms through which psychedelics have long-term effects. No research is perfect, and imperfect designs may still yield useful answers. However, to avoid spurious findings and over-stated claims that might steer the field in a misguided direction, I humbly suggest future work on psychological mechanisms of psychedelics, and indeed all drugs, does its best to adhere to the following recommendations:

1. Clearly specify whether studying putative acute or post-acute mechanisms through which drugs have their effects or conditions or factors on which their effects depend.
2. Measure relevant variables several, preferably at least three, times. Exercise great caution in analyzing and interpreting cross-sectional data.
3. When conducting mediation analyses, use modern statistical methods, preferring bootstrapping, SEM, latent variables, and a model comparison approach, when possible.
4. Examine the possibility of interactive effects of treatment and mechanism on the outcome and confounders in the mechanism to outcome relation. Consider migrating to the causal mediation analysis framework.
5. Base analyses of mechanisms on strong theoretical grounds and pre-register them.
